# Thermally Reconfigurable Metasurfaces: From Linear Wavefront Control to Nonlinear and Chemical Functionality

**DOI:** 10.1021/acs.nanolett.6c02092

**Published:** 2026-07-10

**Authors:** Omer Can Karaman, Gopal Narmada Naidu, Agostino Di Francescantonio, Diana Dall’Aglio, Elif Nur Dayi, Gloria Davidova, Giulia Tagliabue

**Affiliations:** Laboratory of Nanoscience for Energy Technologies, STI, École Polytechnique Fédérale de Lausanne, 1015 Lausanne, Switzerland

**Keywords:** thermo-optical metasurfaces, reconfigurable nanophotonics, photothermal nonlinearity, resonant dielectric nanostructures, phase-change materials, nonlinear and quantum metasurfaces

## Abstract

Resonant metasurfaces concentrate light into subwavelength volumes where even modest temperature changes can shift, reshape, or extinguish spectral features—turning heat into a powerful control knob. This Review presents a unified perspective on thermo-optical metasurfaces, showing how photothermal transduction and temperature-dependent refractive indices connect a single physical mechanism to diverse functionalities. We trace this connection from wavefront and spectral control in the linear regime to bistability, nonreciprocity, and modulation of nonlinear and quantum optical processes. We further highlight emerging applications at the interface of nanophotonics and chemistry, where thermally generated temperature fields and tunable resonances can control reaction environments, enhance infrared molecular signatures, and switch polaritonic coupling. By comparing material platforms and resonance architectures, we identify key opportunities and challenges that will guide the development of thermo-optical metasurfaces for reconfigurable photonics, spectroscopy, and chemistry.

## Graphical abstract

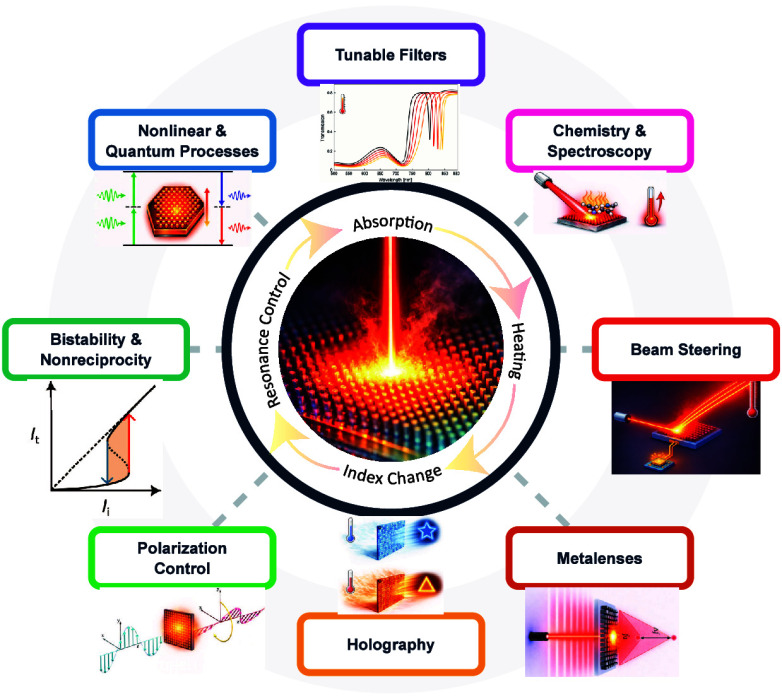

Metasurfaces provide subwavelength control over optical amplitude, phase, propagation direction, and polarization, enabling compact flat-optical components.^1^ As the field moves from static optics toward adaptive and multifunctional devices, postfabrication reconfigurability has become a central challenge. Several tuning routes have been developed, including carrier and electro-optic modulation, liquid-crystal integration, micro–electro–mechanical systems actuation, electrochromic switching, and phase-change materials (PCMs). Thermo-optical metasurfaces occupy a complementary position in this landscape: rather than avoiding heat as a parasitic byproduct of absorption, they deliberately use temperature as a control parameter for reshaping resonant optical responses.

Thermo-optical control is attractive due to the key possibility of contactless, light-based modulation across a wide range of time scales, from picoseconds up to steady state. It is also broadly compatible with dielectric, plasmonic, phase-change, polymer, and hybrid platforms and can be driven globally by external heaters, locally by integrated microheaters, or contactlessly through photothermal absorption. A temperature-induced change in the complex refractive index can shift a resonance, modify its line width or contrast, and thereby reconfigure spectral, wavefront, polarization, and nonlinear-optical functionality. The same absorption–heating–resonance-control loop can also be exploited beyond conventional flat optics: the thermally generated temperature field can locally define reaction environments, while thermally tunable resonances can enhance molecular signatures or switch light–matter coupling conditions. In this way, chemical, spectroscopic, and strong-coupling functionalities are direct extensions of the same thermo-optical mechanism rather than a separate application area. While early thermally tunable metasurfaces were often associated with large-index-contrast phase transitions, recent work shows that even modest thermorefractive coefficients can yield strong modulation when amplified by high-*Q* resonances, quasi-bound states in the continuum, guided modes, or engineered photothermal transduction.

This Review organizes these developments around a common photothermo-optical feedback loop: absorption generates localized heating, heating perturbs the complex refractive index, and the resulting resonance shift or reshaping changes the optical response (Figure 1). Section 2 introduces this framework and the line-width-normalized metrics used throughout the Review: the resonance detuning normalized to the optical line width, η, its temperature-normalized form, *F*_T_, and the corresponding absorbed- and input-power-normalized efficiencies, $F_{P}^{abs}$ and $F_{P}^{in}s$ , which allow a comparison across different heating modalities. Section 3 and 4 survey material platforms and coupled electromagnetic–thermal design trade-offs. Sections 5–7 then review applications in linear wavefront and spectral control, nonlinear and quantum processes as well as emerging chemical, spectroscopic, and strong-coupling functionalities. By introducing these latter topics already within the common feedback loop, we emphasize that chemistry is addressed here through the same thermally programmable metasurface physics: controlled heat generation, resonant field localization, and temperature-dependent optical response. Finally, the Outlook identifies key challenges for the field spanning performance reporting, nanothermometry tools, thermal packaging, and system integration.

**Figure 1:**
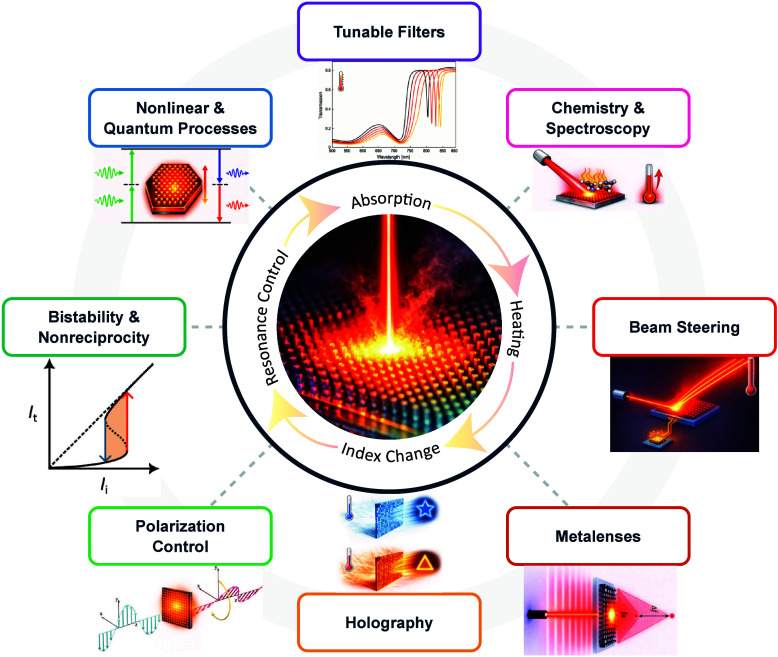
Overview of thermo-optical metasurfaces as a unifying platform for reconfigurable flat optics. At the center, the fundamental photothermo-optical feedback loop is illustrated: optical absorption generates localized heating, which modifies the complex refractive index and, in turn, enables resonance control. This common physical mechanism connects a broad range of functionalities, spanning tunable filters, beam steering, metalenses, holography, and polarization control in the linear regime, as well as bistability, nonreciprocity, nonlinear and quantum processes, and emerging applications in chemistry and spectroscopy. The figure highlights the central theme of this review: a single thermo-optical mechanism underpins a remarkably diverse design space of active and reconfigurable metasurface functionalities.

## Theoretical Frameworks of Photothermal and Thermo-optical Effects

Thermo-optical (TO) metasurfaces transduce a thermal stimulus, externally applied or optically triggered, into a controllable change of phase, amplitude, or polarization of the transmitted, reflected, or generated light by leveraging (i) thermal transport that sets the spatiotemporal temperature field Δ*T*(**r**,*t*) and (ii) the temperature dependence of the complex refractive index that perturbs resonant meta-atoms and collective metasurface modes.^2^ This section provides the physics framework that underpins the application sections.

### Thermo-optic Perturbation: Complex Index as the Control Knob

For a given temperature change Δ*T*, the complex refractive index ñ=n+ik can be linearized by retaining the first-order term of a Taylor expansion:

1 $$\Delta ñ\left(r,t\right)=\left(\frac{dn}{dT}+i\;\frac{dk}{dT}\right)\Delta T\left(r,t\right)$$

equivalently Δε = (∂ε/∂*T*)­Δ*T*.^2^ This approximation is generally suitable for thermorefractive tuning when d*n*/d*T* and d*k*/d*T* vary slowly over the operating temperature range. In contrast, higher-order terms or a state-dependent ñ(T) are required for materials with strongly nonlinear, abrupt, or hysteretic temperature-dependent optical constants, such as PCMs. For resonant metasurfaces, eq 1 leads to two generic actuation channels: (i) *spectral translation* via Δ*n* (shift of the resonance frequency) and (ii) *spectral sculpting* via Δ*k* and/or a thermally modified radiative leakage rate (line width and contrast changes). This “shift + reshape” decomposition provides a compact physical language that applies from thermo-optical switches to nonlinear optics.

### Photothermal Transduction and Thermal Transport

In photothermal operation, absorbed power density *Q*_abs_(**r**,*t*) drives the temperature field via

2 $$\rho c_{p}\;\frac{\partial T}{\partial t}=\nabla \cdot \left(k\nabla T\right)+Q_{abs}\left(r,t\right)$$

where ρ, *c*_p_, and *k* are the density, heat capacity, and thermal conductivity.^2^ Equation 2 represents the conventional Fourier heat-diffusion framework widely employed throughout the thermo-optical metasurface and thermoplasmonics literature. While this continuum treatment does not capture all nanoscale thermal phenomena exactly, it has been extensively and successfully used to model thermal effects even in deeply subwavelength nanophotonic and plasmonic systems. For spatially programmable metasurfaces, the key constraint is thermal cross-talk, conveniently captured by the heat diffusion characteristic length over a time scale τ,

3 $$L_{D}\left(\tau \right)∼\sqrt{\alpha \tau },,\alpha =\frac{k}{\rho c_{p}}$$

which sets the characteristic lateral spread of Δ*T* during modulation or switching.^2^ Equation 3 makes explicit the core trade-off: faster operation (smaller τ) and/or lower diffusivity reduces cross-talk, while strong confinement of *Q*_abs_ increases local Δ*T* for a given absorbed energy.

At the same time, deviations from purely diffusive transport may emerge when characteristic dimensions approach phonon mean-free paths or under ultrafast and strongly localized excitation. In such regimes, non-Fourier effects including ballistic or quasi-ballistic heat transport, interfacial thermal resistance, and nonequilibrium carrier–phonon dynamics may become relevant, making the notion of a single equilibrium temperature less rigorously defined. Exploring non-Fourier thermal transport and nonequilibrium nanoscale heating in metasurfaces therefore remains an important open research direction that may provide additional opportunities for controlling thermo-optical modulation beyond conventional diffusive heat engineering.

### Thermo-optical Nonlinearity, Self-Action, and Nonreciprocity

Under optical pumping, eq 1 gives rise to an effective Kerr-type nonlinearity *n*(*I*) = *n*_0_ + *n*_2_ *I*,^3^ where the thermo-optical contribution to *n*_2_ is set by d*n*/d*T* as well as the pump-to-temperature conversion efficiency. This TO nonlinearity dominates under continuous-wave and long-pulse illumination, where absorbed energy accumulates over many optical cycles. By contrast, electronic nonlinearities, namely, instantaneous two-photon absorption and the picosecond-to-nanosecond free-carrier dispersion it generates, become competitive for ultrashort pulses or low average powers where steady-state heating is suppressed.

In resonant structures, the interplay between field enhancement and the resulting index change creates a feedback loop capable of inducing self-action on intense optical fields. Absorption-driven heating increases *n* (since typically d*n*/d*T* > 0), redshifting the resonance frequency ω_0_ and thereby modifying the intracavity field that drives further heating. Coupled-mode theory captures this feedback as a third-order algebraic equation in mode amplitude that, under red-detuned pumping and for certain ranges of input intensity and loss, admits three solutions, two stable and one unstable, giving rise to optical *bistability* with characteristic S-shaped input–output curves and hysteresis.^4,5^ The two stable “hot” and “cold” states correspond to equilibrium temperatures found by balancing photothermal heat inflow with dissipation.^6,7^

A further consequence of TO nonlinearity is *nonreciprocal transmission*: in geometrically asymmetric resonators, opposite propagation directions produce different field distributions, and a sufficiently strong TO nonlinearity converts these into direction-dependent local permittivities, yielding direction-dependent transmission without external bias or magneto-optical materials.^8^ Because TO nonlinearity is noninstantaneous, this isolation operates as a steady-state effect under exclusive (nonsimultaneous) excitation and requires the thermal time scale to be faster than the illumination dynamics.^8^

Beyond self-action, metasurfaces exploiting high-order optical susceptibilities, including second-harmonic generation (SHG), third-harmonic generation (THG), spontaneous parametric downconversion (SPDC), and spontaneous four-wave mixing (SFWM), rely on engineered resonances,^9,10^ and temperature-induced index changes can modify the resonant conditions governing these processes, affecting conversion efficiency, emission directionality, and spectral characteristics. These nonlinear applications are discussed in Section 6.

### Resonance-Level Description and Figures of Merit

A minimal resonance model treats the thermally tuned mode as a complex pole $\mathrm{\omega ̃}\left(T\right)=\omega_{0}\left(T\right)-i\Gamma \left(T\right)/2$ , where ω_0_ captures resonance translation and Γ captures the optical line width and hence contrast changes. A universal figure of merit for modulation is the detuning normalized to the line width,

4 $$\eta \equiv \frac{\left|\Delta \omega_{0}\right|}{\Gamma }=Q\frac{\left|\Delta \omega_{0}\right|}{\omega_{0}}\approx Q\frac{\left|\Delta \lambda_{0}\right|}{\lambda_{0}}$$

where *Q* = ω_0_/Γ and the last form assumes |Δλ_0_| ≪ λ_0_.^2^ Spectrally, η ≪ 1 leaves the resonance nearly in place relative to its width and produces only small changes at the probe wavelength, whereas η ∼ 1, a line width-scale resonance displacement, translates the resonance by roughly one full width at half-maximum (fwhm), switching a fixed probe between the near-peak and near-tail of the line and yielding strong on/off contrast.

To compare the actuation performance across studies employing different heating modalities, it is useful to distinguish (i) temperature-set point control via external heaters or hot plates from (ii) power-driven control via photo- or electrothermal actuation. For the former, an intrinsic susceptibility metric is

5 $$F_{T}\equiv \frac{\eta }{\Delta T}$$

which quantifies line-width-normalized detuning per kelvin and is directly comparable across platforms independent of heater implementation.

In power-driven schemes, it is useful to separate intrinsic photothermal transduction from system-level power cost by defining two complementary metrics,

6 $$F_{P}^{abs}\equiv \frac{\eta }{P_{abs}},,F_{P}^{in}\equiv \frac{\eta }{P_{in}}$$

where *P*_abs_ = ∫∫∫*Q*_abs_ d*V* is the absorbed power and *P*_in_ is the incident optical power (or electrical input power for electrothermal actuation). The two are related by an effective absorption efficiency *A*_eff_ via *P*_abs_ = *A*_eff_ *P*_in_, so that $F_{P}^{in}=A_{e\mathrm{ff}}F_{P}^{abs}$ .

Together, these metrics highlight the main design levers: maximize resonant leverage (*Q* and modal overlap), maximize photothermal transduction through absorption localization and coupling efficiency, and manage diffusion-limited cross-talk via eq 3.

## Material Platforms

The thermo-optic coefficient d*n*/d*T*, temperature-dependent absorption d*k*/d*T*, thermal conductivity κ, and volumetric heat capacity ρ*C*_p_ collectively determine tuning efficiency, spatial resolution, and temporal response.^11^ Rather than treating material classes as fixed categories, it is more instructive to regard them as design parameters: optical loss governs photothermal heating efficiency, d*n*/d*T* dictates spectral sensitivity, κ controls lateral heat spreading, and ρ*C*_p_ sets the thermal time constant and potential memory effects. Current material platforms span all-dielectric semiconductors, plasmonic systems, PCMs, 2D materials, hybrid architectures, and polymers.^12,13^

These coefficients, however, are not single-material constants but are themselves wavelength-dependent and should be understood as evaluated at the operating wavelength. In semiconductors, the dispersion reflects temperature-induced band-gap narrowing, lattice expansion, and Fermi–Dirac smearing of carrier occupations,^12,13^ with both coefficients growing sharply as the band edge is approached, as exemplified by the large |d*n*/d*T*| of PbTe operated near its 3.9 μm band edge;^14^ in plasmonic metals, it arises from the temperature dependence of Drude damping at long wavelengths and from phonon-assisted broadening of interband transitions near the d-band edges.^15^ The effect is negligible for narrowband operation away from electronic absorption edges but becomes a practical design constraint for broadband or dispersion-engineered metasurfaces such as achromatic metalenses; the accumulated chromatic dispersion of long-haul optical communications does not transfer here because the response is set by the local resonance rather than by propagation-length effects. These wavelength-dependent considerations shape the operating-window choices made across the platforms surveyed below.

### All-Dielectric Semiconductor Platforms

These constitute a widely adopted class of metasurfaces, utilizing semiconductor materials to achieve predominantly dielectric (nonplasmonic) Mie-type resonances with low dissipative loss.^2,16^ To better distinguish between the distinct thermo-optical trade-offs within this category, these platforms can be classified into two primary subclasses.

#### High-Index Semiconductor Platforms

Silicon-based materials, including crystalline silicon (c-Si), amorphous silicon (a-Si), hydrogenated amorphous silicon (a-Si:H), silicon carbide (SiC), and germanium (Ge), provide strong Mie-type resonances and pronounced phase accumulation within subwavelength meta-atoms.^17^ III–V semiconductors such as gallium arsenide (GaAs), aluminum gallium arsenide (AlGaAs),^18–20^ and indium phosphide (InP)^21^ further expand the design space with large d*n*/d*T* values, strong nonlinear-optical susceptibilities, and compatibility with active optoelectronic integration. Chalcogenide glasses (As_2_S_3_, As_2_Se_3_, Ge_*x*_Sb_*y*_Se_*z*_) combine high infrared refractive index with thermo-optic coefficients that can exceed those of conventional dielectrics, enabling pronounced resonance shifts under modest temperature variations.^14,22^ Their low thermal conductivity enhances localized heating and spatial control, although at the cost of slower thermal dissipation, making them particularly attractive for infrared reconfigurable metasurfaces and tunable filters.

#### Low-Index and Wide-Band-Gap Platforms

Complementing high-index systems, lower-index and wide-band-gap dielectrics—including titanium dioxide (TiO_2_) and silicon nitride (Si_3_N_4_)—enable low-loss operation in the visible and near-infrared while retaining moderate thermorefractive sensitivity.^23–26^ Across all of these materials, semiconductor metasurfaces operate in two complementary spectral regimes: below the electronic band gap, where minimal absorption permits ultrahigh-*Q* resonances, and above the band gap, where strong interband absorption can be deliberately engineered to enhance photothermal self-heating.

### Plasmonic Platforms

These typically rely on metals such as Au, Ag, and Cu, whose high free-electron density enables strong localized surface plasmon resonances and field confinement well below the diffraction limit. Unlike semiconductors, the optical constants of metals are dominated by free-electron behavior: increasing temperature enhances electron–phonon and phonon–phonon scattering, leading to increased Ohmic losses, resonance broadening, red shifts, and reduced quality factors.^15,27–30^ These temperature-dependent mechanisms present both challenges, primarily reduced resonance contrast, and opportunities, particularly efficient local heating for photothermal tuning.

We note that thermoplasmonics is a mature field with extensive dedicated reviews and books,^31,32^ whereas this Review focuses primarily on the emerging area of thermo-optical metasurfaces based on dielectric, weakly absorptive, and high-*Q* resonant platforms. Metals are therefore not included in the d*n*/d*T*-based material maps of Figure 2a,b because their thermal optical response is more appropriately described by the temperature-dependent complex permittivity ϵ­(ω,*T*), including changes in Drude damping, interband broadening, Ohmic loss, and resonance line width. In addition, in many thermally tunable plasmonic metasurfaces, the observed resonance shift is governed primarily by thermo-optical, phase-change, or structural changes in the surrounding medium, such as liquid crystals, hydrogels, or VO_2_,^33–36^ rather than by intrinsic metal tunability, making a direct metal-by-metal comparison less representative of the metasurface-level performance.

**Figure 2:**
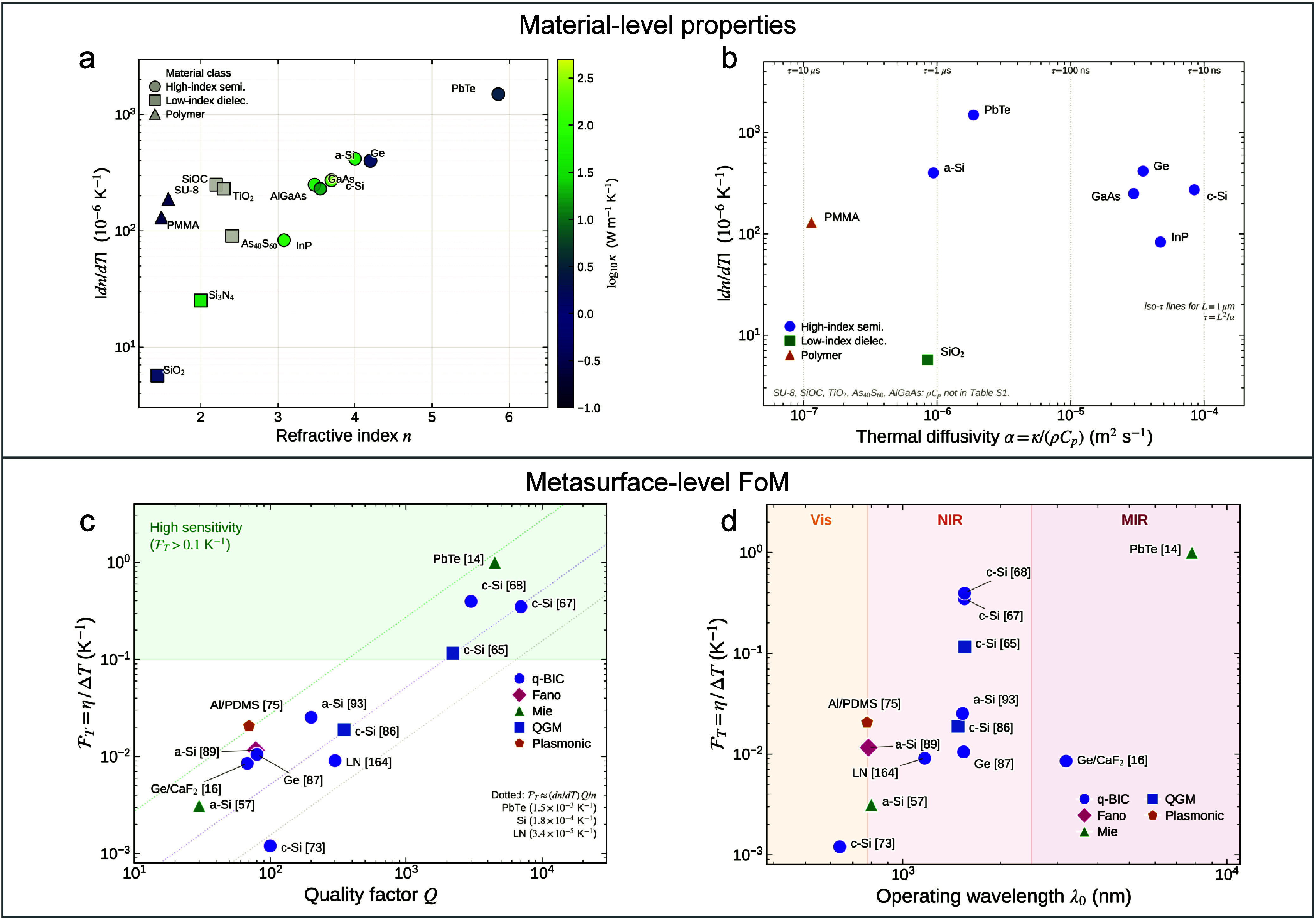
Material-level properties and metasurface-level thermo-optical figures of merit. Material-level properties: (a) Thermo-optic coefficient magnitude, |d*n*/d*T*|, plotted against refractive index *n* for representative high-index semiconductors, low-index dielectrics, and polymers, with color denoting log κ. (b) Thermo-optic coefficient magnitude plotted against thermal diffusivity, α = κ/ρ*C*_p_, with dotted guide lines indicating representative diffusion times for *L* = 1 μm. Metasurface-level figures of merit: (c) Thermal tuning sensitivity, $F_{T}=\eta /\Delta T$ , as a function of the quality factor *Q*, showing the role of resonance line width in thermo-optical reconfiguration. (d) Thermal tuning sensitivity as a function of the operating wavelength λ_0_, comparing representative visible, near-infrared, and mid-infrared metasurface platforms. Panels a and b are based on Table S1, while panels c and d are based on Table S2.

### Phase-Change Materials (PCMs)

These provide large, material-state-dependent refractive index contrast through thermally driven changes of the material state. Unlike purely thermorefractive materials, phase-transition materials undergo thermally driven changes of the optical properties, producing substantial changes in both the real and imaginary parts of the complex refractive index. In nonvolatile chalcogenide PCMs, this typically corresponds to switching between amorphous and crystalline phases, whereas correlated oxides such as VO_2_ exhibit a generally volatile insulator–metal transition. Chalcogenide alloys such as Ge_2_Sb_2_Te_5_ (GST) and related compositions, including GeSbSe and Sb_2_S_3_, exhibit large index modulation in the near- and mid-infrared.^37–41^ Correlated oxides such as VO_2_ display insulator–metal transitions accompanied by dramatic permittivity changes near their critical temperature.^42–44^ A key advantage of nonvolatile chalcogenide PCMs is their intrinsic structural memory: once switched between amorphous and crystalline states, the optical state persists without continuous power consumption, enabling bistable or multilevel operation.^45^ Trade-offs include increased optical absorption in certain phases, finite switching endurance, and the need for carefully engineered heat delivery to control phase transitions reliably.

### Two-Dimensional (2D) Materials

These have emerged as versatile ultrathin active layers or transparent nanoheaters integrated with dielectric or plasmonic resonators. Graphene is particularly attractive due to its high electrical conductivity, broadband optical response, and extremely low thermal mass, enabling efficient Joule heating with minimal perturbation to the underlying optical mode. Transition-metal dichalcogenides (e.g., MoS_2_ and WS_2_) and black phosphorus offer strong light–matter interaction and tunable absorption, although their relatively limited intrinsic thermo-optic index contrast typically necessitates hybrid designs in which the 2D material provides heating or carrier-induced modulation while phase control is maintained by a high-index dielectric resonator.

### Hybrid Architectures

These intentionally decouple optical confinement from heat generation by combining a low-loss high-index dielectric resonator with an absorptive or a conductive layer, such as a plasmonic nanoantenna,^46,47^ a graphene sheet,^48^ or a phase-change film,^49–52^ that provides controlled thermal actuation. This separation enables independent optimization of the resonance quality factor and heating efficiency, overcoming the intrinsic trade-off between strong optical confinement and dissipative loss. By engineering the modal overlap with selectively heated regions, hybrid metasurfaces allow precise thermorefractive control while mitigating unwanted global thermal diffusion, making them particularly attractive for reconfigurable beam steering, tunable lenses, and active wavefront shaping.

### Thermo-optic Polymers

These polymers (PMMA, SU-8, polystyrene, polycarbonate, CYTOP, and liquid crystal polymers) offer comparatively large, typically negative thermo-optic coefficients (d*n*/d*T* ∼ −10^–4^ K^–1^) and low thermal conductivity (0.1–0.3 W m^–1^ K^–1^).^53–55^ In these materials, the thermo-optic response arises from temperature-induced density changes and variations in the molecular polarizability rather than electronic band-structure effects. Their low thermal conductivity enables strong spatial confinement of heat and low-power tuning, although it also limits dissipation speed, and long-term stability is constrained by glass transition temperatures. Polymer-based platforms are therefore well suited to flexible, lightweight, and large-area reconfigurable photonic devices.

Across these material classes, the key design variables are not only the magnitude of the thermo-optic response but also how optical loss, thermal conductivity, and heat capacity jointly determine heating efficiency, confinement, and speed. The same material can be favorable for one application and limiting for another: low loss supports narrow resonances, strong absorption enhances photothermal actuation, low thermal conductivity improves spatial confinement, and thermal memory enables multistate operation. The challenge is therefore not simply to select the “best” material but to match material properties to the desired actuation modality, operating wavelength, and spatiotemporal performance target.

Panels a and b of Figure 2 summarize the resulting design space, plotting the thermo-optic coefficient magnitude |d*n*/d*T*| against refractive index *n* (panel a) and against thermal diffusivity α = κ/ρ*C*_p_ (panel b), with diffusion-time guide lines evaluated for a characteristic length scale *L* = 1 μm, for representative platforms compiled in Table S1. Two complementary trends emerge. First, |d*n*/d*T*| correlates broadly with *n* across material families: polymers and low-index dielectrics cluster at modest sensitivities, silicon and III–V semiconductors form an intermediate plateau, and PbTe stands apart at high index and exceptionally large |d*n*/d*T*|, reflecting its near-band-edge operation at 3.9 μm, as discussed earlier in this section. Second, α varies by nearly 3 orders of magnitude across these same materials, so platforms with comparable |d*n*/d*T*| can differ in the thermal time constant by orders of magnitude: silicon and germanium occupy the ∼10 ns regime for a characteristic length scale *L* = 1 μm, PbTe sits near ∼1 μs, and PMMA approaches ∼10 μs. These two views make the trade-off concrete: silicon and germanium combine moderate |d*n*/d*T*| with the fastest thermal response, PbTe achieves the largest |d*n*/d*T*| at intermediate speed, and polymers compensate for their lower index with strong heat confinement at the cost of much longer time constants, so the choice of material effectively encodes a position on a sensitivity–speed plane that the device application must select.

## Modeling and Design Trade-Offs

The material considerations outlined above translate directly into a modeling problem: how should one predict thermo-optic response when resonance properties, heat generation, and thermal transport are all interdependent? Accurate device design requires coupled electromagnetic–thermal modeling, in which the optical solution determines the absorbed-power distribution and the thermal solution returns the temperature-dependent permittivity that reshapes the optical response. This coupling becomes especially important in resonant metasurfaces, where even modest self-heating can measurably shift narrow spectral features and alter the device performance.^56^

In practice, thermo-optical metasurface modeling is inherently multiphysics and multiscale. Depending on the material platform and excitation regime, the relevant processes may include electromagnetic resonances, photothermal absorption, carrier generation and diffusion, electron–phonon coupling, heat transport, nonlinear-optical feedback, phase transitions, and in certain material platforms, thermomechanical effects such as thermal expansion, stress-induced deformation, or morphology changes. Importantly, these processes span vastly different temporal and spatial scales, ranging from femtosecond optical and carrier dynamics to microsecond-to-millisecond thermal diffusion over micrometer-scale substrates. Consequently, the principal challenge is often not the inclusion of temperature-dependent optical constants alone but the self-consistent treatment of the multitude of coupled processes that ultimately determine the temperature-dependent permittivity and therefore the optical response. In this context, multiphysics modeling approaches based on finite-element method and finite-difference time domain frameworks, implemented in widely used platforms such as COMSOL Multiphysics and Ansys Lumerical, have become indispensable tools for practical thermo-optical metasurface design. These approaches enable self-consistent coupling between electromagnetic, thermal, carrier transport, and even mechanical simulations, allowing accurate prediction of heat generation, thermal diffusion, and resonance evolution across different material platforms and operating regimes.

Different modeling strategies emerge depending on the relevant temporal regime. Steady-state heat diffusion models are often sufficient for static tuning applications such as reconfigurable lenses or beam steering, where the temperature distribution reaches equilibrium under continuous heating. Dynamic applications such as optical modulation or switching, by contrast, require time-dependent thermal simulations that resolve the evolution of temperature fields and optical resonances across time scales ranging from femtosecond optical dynamics to microsecond-to-millisecond thermal diffusion. In optically driven metasurfaces, thermal modulation often represents the final stage of a cascade of nonequilibrium processes initiated by photon absorption, including charge-carrier generation, carrier diffusion and thermalization, and eventual energy transfer to the lattice through electron–phonon coupling. Consequently, transient refractive-index changes may contain competing contributions from free-carrier, Drude-like, band-filling, and thermal effects. Fourier heat diffusion has been extensively and successfully employed in nanophotonic and thermoplasmonic systems, including deeply subwavelength plasmonic nanoantennas, and generally remains a useful first-order approximation for describing the average temperature evolution and thermo-optical response in metasurfaces.^57^ However, at ultrafast time scales or extreme nanoscale dimensions, the assumption of a local thermal equilibrium can break down. Immediately following optical excitation, energy is first deposited into the electronic subsystem before being transferred to the lattice through electron–phonon coupling. In this regime, two-temperature models (TTMs) are commonly employed, particularly for plasmonic and strongly absorbing materials, to separately describe electron and lattice temperatures and their nonequilibrium dynamics.^58^ Semiconductor metasurfaces driven near or above the band gap may additionally require carrier diffusion and recombination dynamics, motivating the use of inhomogeneous two-temperature models (I2TM) and related carrier-transport frameworks.

A central aspect common to all of these modeling approaches is the treatment of temperature-dependent optical properties. Regardless of whether the underlying physical processes originate from thermal diffusion, carrier dynamics, phase transitions, nonlinear feedback, or thermomechanical effects, thermo-optical modulation ultimately manifests through temperature-dependent changes in the complex permittivity. Consequently, the accurate incorporation of temperature-dependent material parameters remains fundamental across essentially all thermo-optical metasurface platforms. For periodic metasurfaces, effective medium approximations can provide useful intuition about thermal shifts in resonance frequencies. Strongly resonant structures, particularly those supporting high-*Q* modes or sharp Fano resonances, require full-wave simulations that resolve the spatial distribution of both optical fields and temperature gradients within individual metaatoms.

Analytical approaches complement full numerical simulations by providing physical intuition and computational efficiency. Coupled-mode theory offers a convenient framework for describing how temperature-induced permittivity changes shift the resonance frequency and line width. In this description, the resonance shift depends on the spatial overlap between the temperature-induced permittivity perturbation and the optical field distribution, making modal confinement, absorption distribution, and overlap with heated regions critical design factors for maximizing tuning efficiency while minimizing undesired line width broadening.^59,60^

More recently, inverse-design and topology-optimization approaches have emerged as powerful alternatives for navigating the large thermo-optical design space. Rather than manually optimizing the geometry, these methods iteratively determine spatial material distributions that maximize targeted optical and thermal objectives simultaneously. Yoon et al. applied topology optimization to transient heat conduction in thermo-optic silicon modulators,^61^ while Rogers et al. demonstrated thermo-optic-enabled topology optimization for ultracompact tunable photonic devices.^51^ Similarly, Naidu et al. introduced inverse thermal design strategies for engineering prescribed nanoscale temperature landscapes in dielectric metasurfaces.^62^ These approaches illustrate how the thermo-optical metasurface design is increasingly evolving from forward simulation to algorithmically assisted multiphysics optimization.

The architecture used to deliver heat plays an equally central role. Heating can be implemented globally via external stages or hot plates, locally through integrated resistive microheaters or transparent conductive layers such as indium–tin oxide or graphene, or optically through resonant photothermal absorption. Each approach introduces different spatial and temporal heating profiles that influence the tuning efficiency, switching speed, and power consumption. Integrated heaters enable localized and programmable temperature control but add fabrication complexity, whereas optical pumping provides fast heating that may suffer from nonuniform temperature distributions due to collective heating effects.^59,63^

These considerations define a set of fundamental design trade-offs. Increasing optical absorption improves the heating efficiency but can degrade resonance quality factors and spectral selectivity. Materials with low thermal conductivity allow strong spatial confinement of heat but slow cooling and switching. High thermo-optic coefficients enhance tuning sensitivity but may introduce thermal instability in narrowband resonances. Ultrahigh-*Q* modes maximize spectral sensitivity but may respond more slowly due to increased thermal inertia. Successful designs therefore require simultaneous optimization of optical resonances, heat generation pathways, and thermal diffusion, treating the metasurface as a fully coupled multiphysics system.

## Dynamic Wavefront and Spectral Control in the Linear Regime

With the physical framework and material design space established, we now survey experimental demonstrations of thermo-optical metasurfaces operating in the linear regime, where the temperature-induced index perturbation is small enough that the resonance response is well described by the linearized figures of merit η, $F_{T}$ , and $F_{P}$ introduced in the “Resonance-Level Description and Figures of Merit” section. Applications in this regime span tunable spectral filters, reconfigurable beam steering, varifocal metalenses, metaholography, and polarization synthesis. To contextualize the performance landscape before discussing each in turn, Table S2 compiles representative resonance platforms and their thermo-optical metrics, with Figure 2c,d providing a graphical summary.

Several trends emerge from Table S2 and Figure 2c,d. First, the thermal tuning sensitivity $F_{T}$ increases with *Q* but not linearly: quasi-BIC and guided-mode platforms reach $F_{T}$ ∼0.1 – 0.4 K^–1^ at *Q* > 10^3^, whereas Mie resonances with *Q* ∼ 30 achieve $F_{T}$ roughly 2 orders of magnitude lower, reflecting the direct *Q* leverage encoded in eq 4. Second, mid-infrared platforms stand out for their power-normalized performance: PbTe meta-atoms^14^ combine an exceptionally large thermo-optic coefficient with high *Q*, yielding $F_{P}$ values several orders of magnitude above any visible or near-IR entry. This suggests that the mid-IR, where many materials exhibit both large d*n*/d*T* and low absorption loss, remains an underexploited spectral window for power-efficient TO devices. Third, the distinction between $F_{P}^{abs}$ and $F_{P}^{in}$ proves informative, where both are reported in Table S2: the ratio $F_{P}^{in}/F_{P}^{abs}$ directly yields *A*_eff_, which varies from near-unity for PbTe operating above the band gap to ∼0.2–0.5 for dielectric metasurfaces pumped near resonance,^57,65^ quantifying how much of the incident power budget is actually transduced into a thermal perturbation. The bistability entries at the bottom of the table extend the comparison into the nonlinear self-action regime, spanning 3 orders of magnitude in *Q* (from ∼4 in single Mie nanocuboids^66^ to ∼600 in quasi-BIC supercavities^4^). Crucially, the same figures of merit acquire a qualitatively different character when Δλ_0_ is self-induced rather than externally imposed: resonance shifts and heating are coupled through the feedback loop of the Introduction section, so the listed η and $F_{T}$ values represent operating-point-dependent quantities rather than intrinsic material-resonance susceptibilities. Finally, it is notable that power-normalized metrics remain unavailable for the majority of entries because most studies employ external heaters rather than photothermal or electrothermal actuation. This gap underscores the need for standardized reporting conventions.

With this performance landscape in view, we now turn to the linear regime applications it enables, beginning with tunable filtering and spectral shaping.

### Tunable Filters and Spectral Shaping

Thermo-optical metasurfaces are especially effective for filtering and spectral shaping when governed by a sharp resonance because even a small thermally induced perturbation in Δ*n* or Δ*k* can produce a line-width-scale spectral shift or contrast change.^67–69^ Panels c and dFigure [Fig fig2] benchmark representative platforms from Table S2: panel c plots $F_{T}$ against *Q*, showing that high-*Q* resonances reach line-width-scale shifts at modest Δ*T*, while panel d maps $F_{T}$ from the visible to the mid-infrared, highlighting how spectral coverage tracks the choice of material platform. This high-*Q* sensitivity arises because a given thermo-optic resonance shift corresponds to a larger fraction of the line width. It is particularly pronounced in weakly radiative Fano-like modes, including quasi-BIC and so-called quasi-dark metasurfaces, where suppressed radiative leakage gives rise to narrow spectral features suitable for notch and bandpass operation.^67,68^ In a representative quasi-dark silicon platform, the reported tuning slope of ∼0.204 nm/K illustrates how narrowband filtering can be both sensitive to thermal drift and useful for low-energy reconfiguration.^68^

Thermally induced material perturbations have also been proposed to realize quasi-BICs with continuously tunable *Q*, enabling high-contrast switching when the thermal shift approaches the resonance fwhm.^69^ Nonlocal metasurfaces based on quasi-guided modes provide a complementary route: thermo-optic modulation in silicon has been experimentally demonstrated at *Q* ≈ 2200 with >55% modulation depth under weak 532 nm continuous-wave pumping (<4 W/cm^2^), corresponding to only ∼10 K equivalent heating, with similar behavior verified electrically.^65^ Lithium niobate offers a further nonlocal platform for reversible thermal tuning without high-voltage electro-optic drive.^70^

High-*Q* resonances can also enable thermally tunable filtering with small refractive-index changes. In spatially addressable high-*Q* transmissive metasurfaces (e.g., a-Si pillar platforms with *Q* up to ∼9800), the index change required for programmable spectral masking reduces to Δ*n* ∼ 2.6 × 10^–3^ and microsecond-scale reconfiguration (e.g., ∼7.3 μs) becomes achievable, with realistic heater designs providing a path from single filters to programmable spectral masks.^71,72^

Alternatively, loss-dominated control can yield “spectral deletion”: in visible-band resonant silicon metasurfaces, photothermal heating that increases absorption (reported as Δ*k* < 0.05) can quench a q-BIC and produce >80% reflectance modulation, with Raman thermometry indicating that temperature rises to ∼285 °C under MW/cm^2^-scale pumping.^73^ Wavelength-separated control further mitigates the absorption–throughput trade-off by engineering strong absorption at a control wavelength while preserving transmission at the signal wavelength, for example, via dipole–quadrupole interference.^74^ Beyond narrowband filters, multispectral absorber designs extend thermo-optic reconfiguration across broad UV–near-IR ranges, and PCM-loaded resonant cavities enable multistate spectral control.^75,76^

Thermo-optical spectral control can also operate dynamically. In modulated photothermal excitation of dielectric metasurfaces, the optical response time can be substantially faster than the thermal time constant (τ_opt_ ∼ 0.5 μs vs τ_th_ ∼ 3.5 μs), yielding nontrivial transient trajectories and even effective frequency doubling of the output modulation under appropriate detuning (Figure 3a).^57^ Such an operation requires careful thermometry because Raman-based temperature readout can itself be altered by resonance-enhanced excitation and emission and by Purcell-mediated changes in the Raman efficiency.^77^ Taken together, thermo-optical spectral devices can operate through resonance shifts, line-width reshaping, loss-mediated suppression, and transient dynamics across dielectric, nonlocal, and hybrid platforms.^65,67,69,73,74^

**Figure 3:**
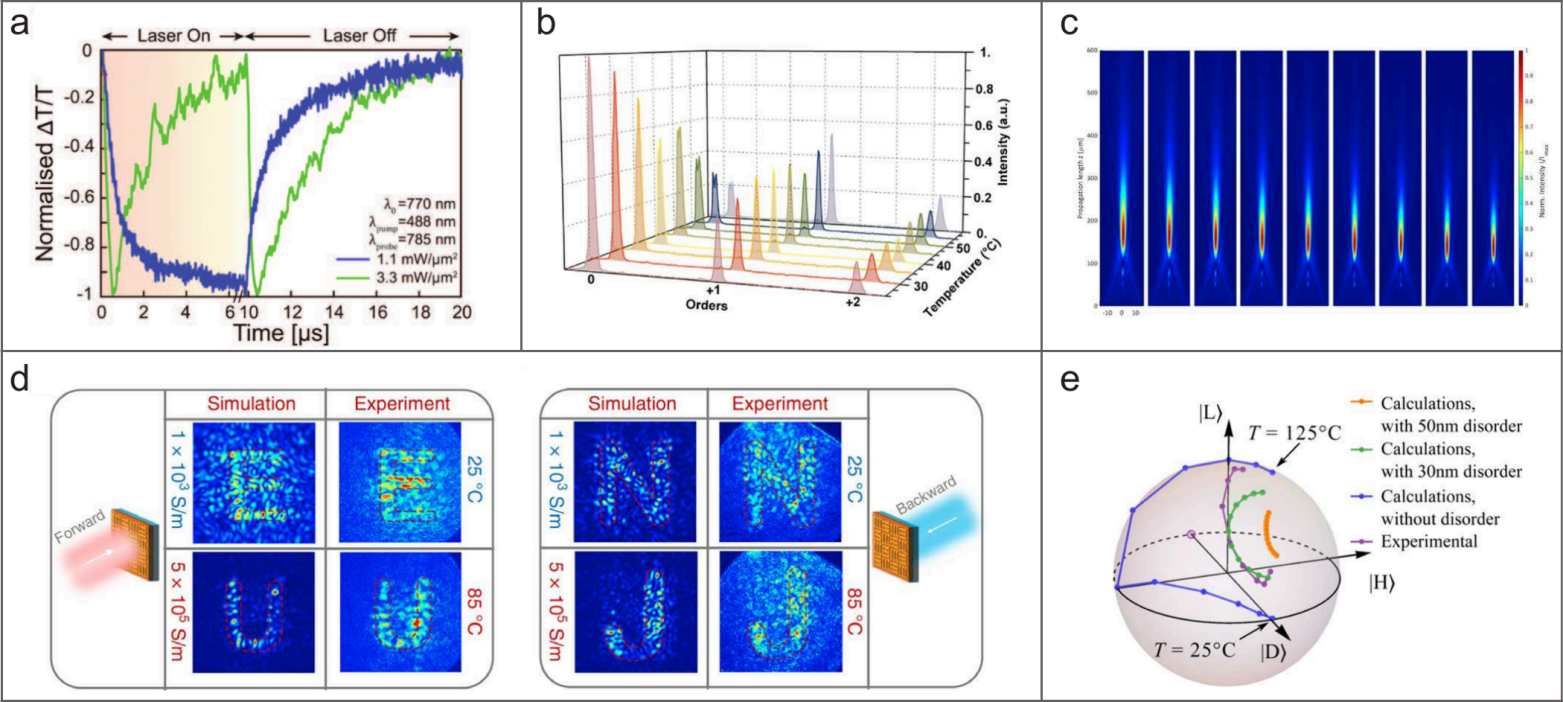
Dynamic wavefront and spectral control enabled by thermo-optical metasurfaces. (a) Transient thermo-optical spectral dynamics in a dielectric metasurface under photothermal excitation, showing distinct heating and cooling trajectories associated with detuned resonant probing. Adapted from ref 57. (b) Thermally controlled beam switching in a liquid-crystal-infiltrated dielectric metasurface, showing the temperature-dependent redistribution of transmitted intensity among the zeroth, first, and second diffraction orders as the liquid-crystal transitions from the nematic to the isotropic state. Adapted from ref 54. (c) Thermally reconfigurable metalens operation, showing simulated beam propagation through a silicon metalens at increasing temperatures and the corresponding shift of the focal plane. Adapted from ref 64. (d) Thermally active Janus metaholography, where the reconstructed holographic image depends jointly on the illumination direction and temperature. Adapted from ref 44. (e) Thermo-optic polarization control visualized on the Poincaré sphere, showing continuous evolution of the output polarization state with temperature. Adapted from ref 16.

### Beam Steering and Deflectors

Thermo-optical metasurfaces enable reconfigurable beam steering by rewriting the phase gradient across an aperture through temperature-dependent material responses. Existing approaches fall into three categories.

(i) *Environment-mediated tuning*, where heating modifies an embedding layer: Komar et al. operated at λ = 745 nm and thermally redistributed the transmitted power between the zeroth and first diffraction orders in a liquid-crystal-infiltrated dielectric metasurface. The total transmitted power decreased by only ∼20%, while the switching contrast between the two orders, quantified as half the difference between the off- and on-state zeroth/first-order intensity contrast, reached 0.48 on a scale where unity represents complete order switching (Figure 3b).^54^

(ii) *Meta-atom-mediated tuning*, where thermo-optic index changes shift resonant pixel phases: Horie et al. reported a tuning efficiency of ∼0.77 nm/mW with ∼70 μs response times, demonstrating θ_max_ ≈ ±1.7° deflection at λ = 1550 nm using a 6 × 6 array (pitch *p* = 26 μm), with up to ∼40% deflection efficiency and ∼8% nearest-neighbor thermal cross-talk.^78^ Complementary design studies have mapped how programmable phase states translate into anomalous-reflection targets, while explicitly quantifying the impact of phase quantization and amplitude–phase coupling on steering efficiency and side-lobe levels.^79^ Thermo-optical rephasing can also steer generated beams, such as opto-thermally steered second-harmonic emission from dielectric metastructures.^19^

(iii) *Material-state tuning* via PCMs extends steering to multilevel, potentially nonvolatile operation: GST pixel designs target phase-only steering at telecom wavelengths;^37^ In_3_SbTe_2_ reflective gratings steered to 46° at λ = 8.6 μm with up to 75% anomalous reflection;^80^ VO_2_ coding metasurfaces produced discrete steering states up to ∼52° at 0.6 THz with broadband angle dispersion.^42^ Control–signal decoupling via mode- and wavelength-selective absorption engineering mitigates photothermal throughput penalties.^74^

### Varifocal Metalenses

Thermo-optical metalenses exploit temperature-dependent material responses to reconfigure the wavefront while keeping a planar lithographically defined aperture. TO tuning strategies fall into three categories.

For *continuous* TO tuning, Iyer et al. analyzed uniform heating of a high-index dielectric metalens and showed that a thermally induced modal index shift of Δ*n* ∼ 0.15 (from InSb Mie resonances) can meaningfully shift the lens operating point, demonstrating tunable focal behavior (e.g., *f* ≈ 45 μm) and a sizable shift of the optimal focusing wavelength (Δλ_f_ ≈ 500 nm).^81^ In the visible, Archetti et al. demonstrated a heater-compatible route for thermoreconfigurable silicon metalenses by combining a geometric phase with a temperature-dependent resonant phase shift: at λ = 632 nm, they report a continuous, near-linear focal-length modulation of ∼21% (from 165 μm at 20 °C to 135 μm at 260 °C) with diffraction-limited quality (Strehl ratio ≈ 0.99) and an average conversion efficiency of ∼26%^64^ (Figure 3c). Temperature can also act as an unwanted perturbation: Lee and Kyoung discuss how thermal drift impacts focal shift and aberrations, motivating thermal-aware design and heat-sinking strategies for stable imaging.^82^

*PCMs* provide larger optical contrast and enable stronger varifocal or switchable behavior, often in discrete states. Bai et al. designed a GST metalens at 1.55 μm where amorphous/crystalline switching yields strong efficiency contrast (∼32.8% vs ∼2%) while keeping the focal length nearly unchanged, enabling “focus on/off” operation.^83^ Qin et al. used Sb_2_S_3_ in a multilayer PB-phase design and demonstrated two-state varifocal behavior at 1310 nm with high simulated focusing efficiencies.^40^ Experimentally, Wang et al. demonstrated ultralow-loss Sb_2_Se_3_ varifocal metalenses with focal-length switching from 41 to 123 μm and ∼95% intensity modulation in a related single-focus design.^41^ In the mid-IR, Tan et al. used GSST for polarization-controlled varifocal metalenses, illustrating how lower-loss PCM choices and polarization multiplexing expand functionality in longer-wavelength bands.^84^ Related thermoswitching functionality has also been demonstrated via VO_2_ phase-transition physics in metaduplex lens concepts.^85^

*Resonance-gated metalenses* leverage the fact that small TO shifts can move a narrow resonance in or out of alignment with a fixed illumination wavelength, yielding large functional contrast at modest Δ*n*. Klopfer et al. demonstrated a thermally controlled zone-plate resonance-gated lens with measured *Q* up to ∼1350; at moderate *Q* ∼ 350, a ∼50 °C change shifts the resonance by one line width, enabling strong amplitude switching.^86^ Malek et al. similarly demonstrated quasi-BIC-based thermally switchable metalenses with on/off and multistate focusing.^87^ Thermotunable metalenses have also been pursued in on-chip geometries using inverse-designed silicon metalines, where temperature changes are mapped directly onto focal-length shifts.^88^

Comparing the three strategies reveals complementary strengths and trade-offs. Continuous TO tuning offers smooth, reversible focal-length modulation with diffraction-limited quality^64^ but is limited by the modest thermorefractive index change of conventional dielectrics (Δ*n*/*n* ∼ 10^–3^–10^–2^), typically yielding focal shifts of tens of percent over ∼100–250 °C windows. PCM-based designs access much larger index contrast and can switch between well-separated focal states without continuous power consumption^40,41^ but at the cost of discrete operation, finite cycling endurance, and precise thermal control. Resonance-gated metalenses occupy an intermediate niche, exploiting narrow spectral features for large on/off contrast at modest Δ*T*,^86,87^ yet remain best suited for narrowband applications. Across all three approaches, reported efficiencies below ∼30% point to co-optimization of phase coverage, resonance engineering, and thermal management as a key direction for practical varifocal devices.

### Metaholography and Image Switching

Thermo-optical metasurfaces extend holography from static wavefront shaping to thermally addressable image generation. Two mechanisms dominate: continuous tuning of resonant phase and amplitude responses for contrast control and discrete switching of material states such as VO_2_ or PCMs for multi-image holography.

In dielectric platforms, encoding images in spectrally sharp resonances makes modest thermal shifts highly effective at a fixed illumination wavelength. Zangeneh Kamali et al. demonstrated reversible image-contrast inversion using Si resonators with narrow Fano features (fwhm ≲ 10 nm) around ∼772–784 nm by sweeping temperature up to ∼125 °C, passing through a near-zero-contrast intermediate state.^89^ Beyond steady-state control, microsecond-scale optical dynamics in high-*Q* a-Si metasurfaces (τ_opt_ ∼ 0.5 μs vs τ_th_ ∼ 3.5 μs) suggest routes toward time-multiplexed displays.^57^

Phase-transition materials enable high-contrast metaholography by mapping distinct material states to distinct images. In the terahertz range, Liu et al. integrated VO_2_ into resonant metasurfaces and demonstrated temperature-dependent hologram switching across a 0.6–1.0 THz operating band.^90^ Chen et al. introduced a thermally active Janus terahertz metasurface where the incident direction and temperature jointly select among multiple images (Figure 3d).^44^ Frequency multiplexing provides a further degree of freedom: Zhao et al. proposed broadband terahertz holography with VO_2_-state-dependent holograms.^43^ At visible wavelengths, VO_2_ nanofin metasurfaces enable binary-phase hologram switching aligned with photothermal actuation.^91^ In the infrared, spatially programmed thermal emission via multilevel laser writing in GST enables position-selective emissivity control with ∼10^–3^ modulation precision.^38^ Temperature thus serves as a global modulation knob, a discrete state selector, or an additional encoding key combined with frequency, direction, or polarization for secure and multiplexed displays.^43,44,89–91^

### Polarization Control

Thermo-optical metasurfaces enable reconfigurable polarization through temperature-dependent birefringence and resonance-enhanced phase retardance, offering either continuous tuning or discrete multistate control. In the near-IR, Bosch et al. demonstrated a thermo-optic polarization synthesizer in which heating tunes a high-*Q* collective resonance of a Ge metasurface and sweeps output states broadly over the Poincaré sphere within a practical 25–125 °C window (Figure 3e).^16^ Phase-change metasurfaces extend this to switchable devices: He et al. reported programmable polarization rotation spanning ∼20° to ∼155° at 1550 nm via PCM state control,^97^ and waveplate-style functionality switching has been designed using thermal PCM metasurfaces.^98^

In the terahertz range, VO_2_-based phase transitions provide large-contrast polarization conversion and bandwidth tuning. Zhao et al. showed thermal expansion of the polarization–conversion bandwidth from dual bands (0.45–0.77 and 0.97–1.2 THz) to an ultrabroadband regime (0.38–1.20 THz), with the polarization conversion ratio reaching ∼95%.^99^ Ren et al. used VO_2_ “digital” states to program polarization angles (e.g., η ≈ 26.3°, 59.9°, and 77.3° at 1.3 THz) and combine polarization coding with beam-channel generation.^100^ Thermal control also modulates more specialized phenomena, including Faraday rotation^101^ and polarization-selective thermal emission.^102^ Finally, polarization can strongly shape photothermal nonlinear response: Wang et al. reported >2 orders-of-magnitude contrast in photothermal nonlinearity between azimuthal and radial cylindrical-vector-beam excitation, enabling reversible scattering saturation and ∼50 nm localization accuracy.^103^

Overall, these examples demonstrate that thermo-optical effects offer a versatile route for reconfiguring linear metasurface components, enabling spectral, angular, focal, holographic, and polarization control through a common-temperature-driven mechanism.

## Thermo-optical Modulation of Nonlinear Processes

As introduced in the previous section, the TO Kerr-type nonlinearity can drive self-action phenomena including bistability and nonreciprocity, while temperature-induced resonance shifts can modulate parametric nonlinear-optical processes. This section surveys experimental and theoretical demonstrations of both regimes.

### Nonlinear Self-Action

#### Bistability in Thermo-optical Nanoresonators

The feedback mechanism described in the introduction section, whereby photothermal redshifts couple back into the intracavity field, produces optical bistability when the system admits two stable equilibria at a given input power. TO bistability was first observed in silicon microring resonators^7^ and photonic crystals,^104^ where high *Q* facilitated experimental observation. Nishida et al.^66^ subsequently demonstrated that optical bistability can arise even in resonators with *Q* < 10 (for a-Si nanocuboids supporting Mie modes, see Figure 4a), dramatically widening the design space. Engineering the radiative losses allows shaping of the hysteresis cycle in terms of critical power, cycle width, and maximum temperature: Barulin et al.^92^ defined the nonlinear critical coupling condition as γ_r_ = γ_nr_/2, corresponding to the minimum power required for bistability, and demonstrated it experimentally by exploiting dispersive guided-mode resonances to tune radiative losses (Figure 4b).

**Figure 4:**
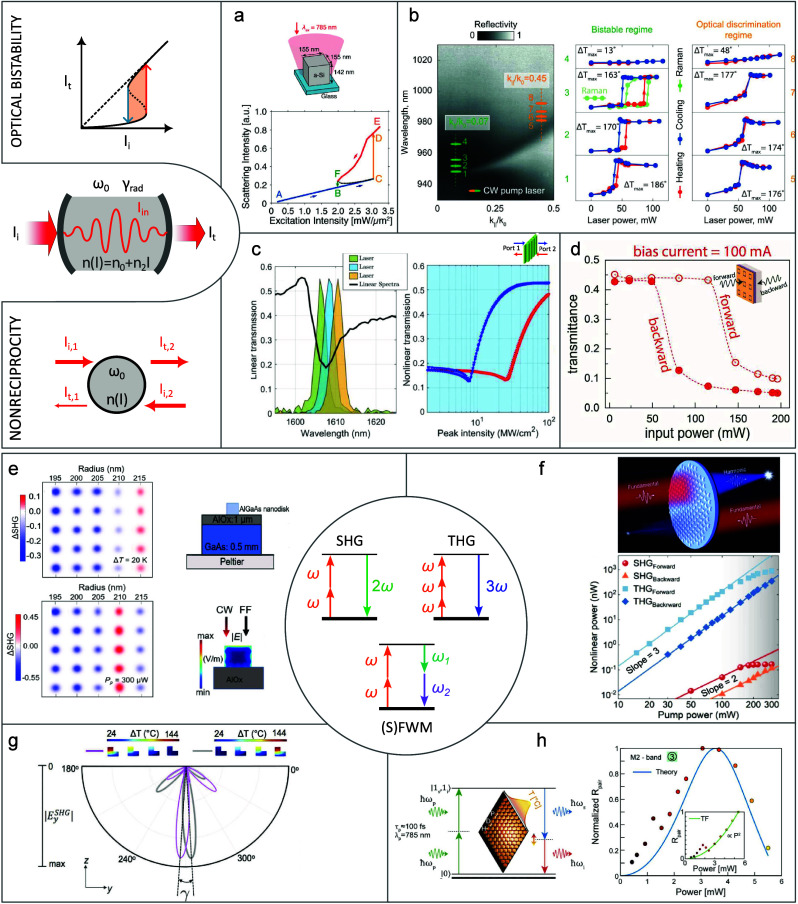
Thermo-optical metasurfaces in the nonlinear regime. (a–d) On the left: illustrations of nonlinear phenomena arising in an optical cavity with resonant frequency ω_0_, radiative losses γ_rad_, and an intensity-dependent refractive index *n*(*I*). At the top: transmitted intensity *I*_t_ as a function of the input intensity *I*_i_, showing the hysteretic behavior typical of bistability. At the bottom: illustration of the nonreciprocal behavior, where the transmitted intensities *I*_t,2_ ≠ *I*_t,1_ for the same input intensity *I*_i1,2_. (a) Hysteretic relationship between the scattered and excitation intensity from a Si nanocube, showing a range where the former differs in the case of increase (B–C–D) or decrease (D–F–B) of the latter. Adapted with permission from ref 66. (b) Influence of the illumination wavelength and transverse wavevector *k*_∥_ on the heating regime of a Si high-*Q* membrane. From the different conditions, identified by the numbered colored spots on the reflectivity map, the corresponding heating/cooling sweeps on the right highlight different temperature hysteresis. Adapted with permission from ref 92. (c) Nonreciprocal transmission (on the right) of a laser from a resonant Si metasurface, where the 1 → 2 transmission (blue curve) differs from 2 → 1 (red curve). On the left: metasurface linear spectrum (in black) and spectrum of the laser used in the nonreciprocity experiment of the right panel (in blue). Adapted with permission from ref 93. (d) Nonreciprocal transmission from a preheated VO_2_ metasurface obtained by sweeping the input power in forward/backward illumination (see the sketch on the top). Adapted with permission from ref 94. (e–h) Thermo-optic tuning of parametric nonlinear processes, sketched in the center, in the classical, and in the quantum regime. (e) Thermo-optically induced variation in the intensity of the second harmonic signal ΔSHG emitted by AlGaAs nanopillars of different radii. The temperature increase is either produced by a Peltier cell (on the top) or by laser illumination (on the bottom). Adapted with permission from ref 18. (f) At the top: nonreciprocal excitation of nonlinear processes (THG and SHG) in a silicon crescent-shaped resonator metalens. At the bottom: THG and SHG emission as a function of the pump power impinging along forward and backward directions. Adapted with permission from ref 95. (g) Theoretically calculated temperature-dependent beam steering of approximately 8° in AlGaAs “nano-chairs”. Adapted with permission from ref 19. (h) Optically induced thermo-optic modulation of SFWM in an a-Si nanodisk metasurface. Adapted with permission from ref 96

The intrinsic state memory of bistable systems can be exploited for volatile thermal memories, where the stored state depends on the heating history. Morsy et al.^5^ demonstrated this in a bistable Si photonic crystal, using intermediate power for readout and high or low inputs for state switching. Volatile thermal memory requires continuous optical pumping; without it, the system thermalizes to the cold state within milliseconds. PCMs are natural candidates for nonvolatile operation. Beyond bistability, coupled optical resonators represent an avenue for multilevel photonic memory.^105,106^

#### Nonlinearity-Induced Nonreciprocity

Building on the mechanism outlined in the introduction section, Cotrufo et al.^93^ experimentally demonstrated a nonreciprocal a-Si metasurface exploiting high-*Q* resonances and TO bistability, reaching ∼10 dB transmission contrast through engineered electromagnetic asymmetry (Figure 4c). Nonreciprocity manifests as a power interval in which the transmissions for opposite propagation directions differ dramatically, with the TO origin confirmed by characteristic hysteresis under reversed power sweep directions. Nonreciprocal behavior was also demonstrated in a VO_2_-based device by King et al.,^94^ where the authors combine the VO_2_ phase transition, tuned by Joule heating, and the nonlinear refractive index to achieve nonreciprocal transmission in the mid-infrared (Figure 4d). An important consideration is that nonreciprocity is a steady-state effect, requiring the nonlinearity dynamics to be faster than the illumination time scale. This is automatically satisfied for instantaneous Kerr nonlinearity but requires additional care for TO effects, whose transient is set by heat generation and diffusion. Hofstrand et al.^107^ addressed this theoretically, demonstrating nonreciprocal pulse shaping arising from a noninstantaneous nonlinearity.

### Thermo-optic Tuning of Optical Nonlinearities

Because nonlinear-optical processes are highly sensitive to resonant overlap and field confinement, thermo-optic tuning provides a direct route to modulating the conversion efficiency, emission directionality, and quantum-photon generation. This control can be achieved through externally applied heating or pump-induced self-heating, depending on the operating regime.

Among the earliest experimental demonstrations, Celebrano et al.^18^ achieved up to 60% SHG modulation from individual AlGaAs nanopillars through ∼40 K heating via a Peltier element or auxiliary optical beam, exploiting thermo-optic shifts of Mie resonances at both fundamental and harmonic frequencies (Figure 4e). Thermal effects can also manifest during nonlinear excitation itself. Qu et al.^108^ investigated SHG from a lithium niobate metasurface and, by monitoring a spectrally distinct resonance during SHG excitation, established a direct correlation between nonlinear emission and real-time resonance shifts. The measurements revealed deviations from the quadratic pump-power dependence expected in the undepleted regime, attributed to thermally induced resonance detuning and highlighting the importance of distinguishing reversible thermo-optic effects from irreversible structural changes at high optical intensities. While analogous TO phase-matching tuning operates in LiNbO_3_ waveguides,^109,110^ metasurfaces provide additional design freedom because the phase-matching condition is relaxed in subwavelength structures.

More complex functionalities have been demonstrated. Yao et al.^95^ showed asymmetric nonlinear wavefront shaping in a silicon crescent metasurface combining Mie resonances with q-BICs: substrate-induced asymmetry produces direction-dependent field enhancement, and as the pump power increases, differential thermal redshifts yield intensity-asymmetric transmission at both fundamental and harmonic frequencies (Figure 4f). TO tuning has also been extended to quantum nonlinear processes. Karaman et al.^111^ demonstrated that pump-induced thermo-optical detuning modulates photon-pair generation via SFWM in a-Si metasurfaces supporting Mie-type electric and magnetic dipole resonances. Unpatterned a-Si films yielded second-order correlations *g* ^(2)^(0) > 400, confirming high-purity nonclassical emission, while the resonant metasurfaces boosted pair rates above 3.8 kHz at submilliwatt pump powers through enhanced nonlinear mode overlap. Crucially, the power-dependent pair rate deviated from the quadratic scaling expected for undepleted SFWM: pump absorption progressively red-shifted the nanodisk resonances, modifying the spectral overlap integral between pump, signal, and idler modes, an effect quantitatively captured by coupled electromagnetic–thermal simulations. This work identifies thermo-optical detuning as a mechanism that must be accounted for, and can potentially be harnessed, in any resonant integrated photon-pair source operating above the material absorption edge^111^ (Figure 4h). TO detuning may further contribute to nonlinear nonreciprocal responses when combined with Kerr-type nonlinearities.^93^

Theoretical work has explored TO-driven reshaping of nonlinear emission patterns. Pashina et al.^112^ investigated a biresonant AlGaAs dimer and showed that temperature-induced changes in the relative spectral overlap produce strongly asymmetric SH radiation patterns. Similarly, Rocco et al.^19^ predicted ∼8° SH beam steering from an AlGaAs “nano-chair” metaunit with modest heating of less than 90 °C (Figure 4g). These studies illustrate how thermo-optic tuning can modulate not only the intensity but also the angular distribution of nonlinear signals.

## Thermo-optical Metasurfaces for Chemistry and IR Spectroscopy

The preceding sections treated temperature as a control parameter for photonic functionality. At the interface of nanophotonics and chemistry, the same thermo-optical coupling acquires an additional role: the localized temperature field generated by resonant absorption can directly influence the surrounding molecular environment, while the thermally tunable resonance remains an optical transducer. In this regime, metasurfaces can locally control reaction environments for photothermal catalysis, enhance and tune infrared molecular signatures for spectroscopy and biomolecular detection, and reversibly move resonances into or out of strong coupling with molecular excitations. This section surveys these emerging directions in the context of photothermal catalysis, infrared spectroscopy, biomolecular detection, and polaritonic chemistry.

### Photothermal Catalysis

Because chemical reaction rates follow the Arrhenius law, $k\propto \exp\left(-E_{a}/k_{B}T\right)$ , even modest local temperature elevations produced by resonant absorption can exponentially accelerate surface reactions, making metasurface-controlled thermal landscapes a powerful lever for heterogeneous catalysis. Un et al.^118^ showed that the nonlinear photothermal response is governed primarily by heat dissipation, particularly the thermal conductivity of the surrounding medium, rather than optical absorption alone, and emphasized the importance of distinguishing thermal from nonthermal mechanisms in hot-carrier catalytic systems.

Motivated by this interplay between heat transport and reaction kinetics, Naidu et al. recently introduced an inverse thermal design framework for a-Si metasurfaces that maps prescribed temperature profiles onto spatially varying nanoresonator geometries,^62^ as shown in Figure 5a. Simulations predict that such engineered landscapes can enhance photothermal reaction rates by more than 30% for gas-phase CO_2_ photomethanation compared to uniform temperature distributions due to the Arrhenius dependence of reaction kinetics even at constant absorbed power and mean temperature.^62^

**Figure 5:**
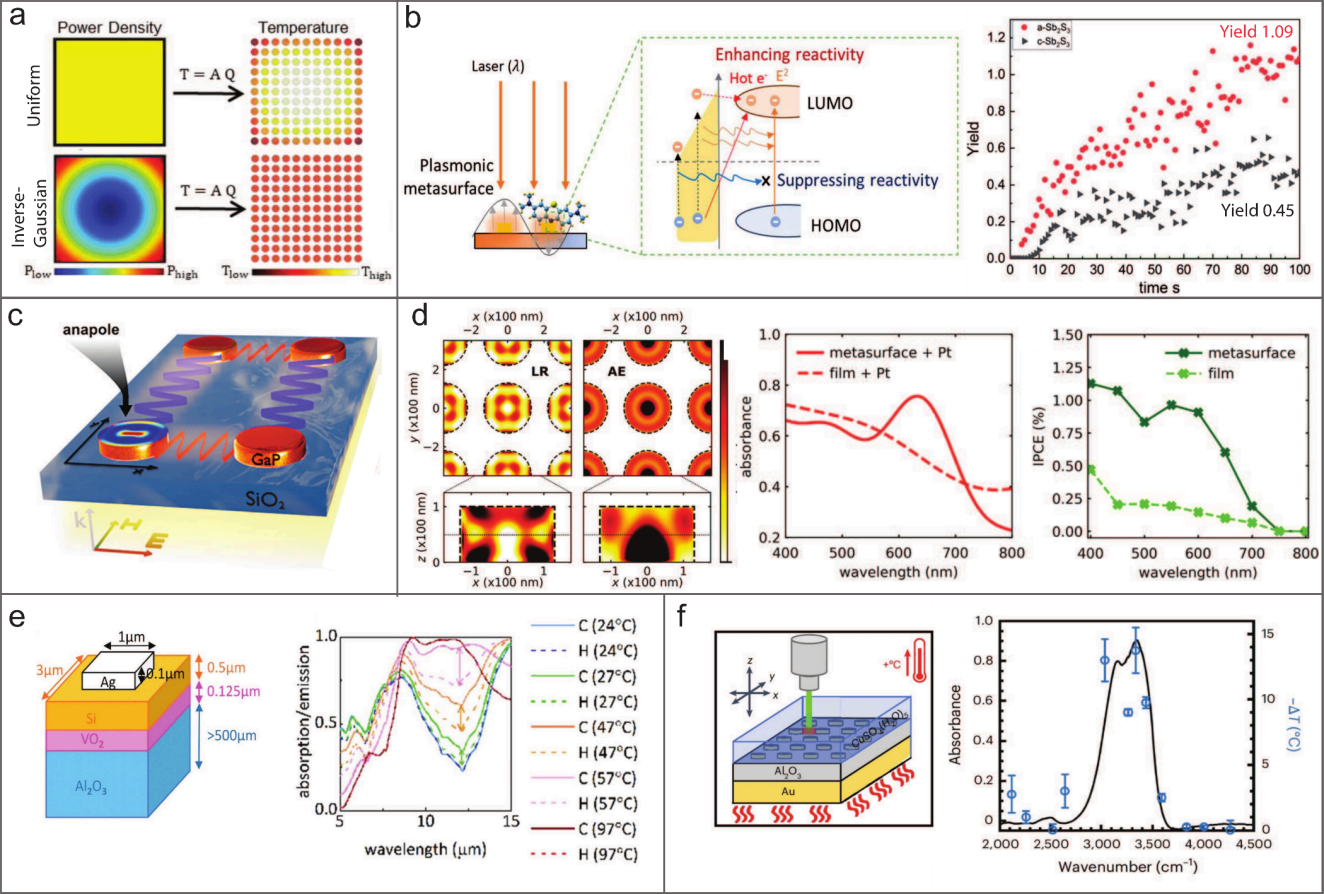
Thermo-optical metasurfaces for photochemistry and infrared applications. (a) Spatially uniform and inverse Gaussian power density profiles (left) and the resulting temperature distributions (right) in amorphous Si metasurfaces, illustrating controlled thermal landscape engineering via tailored optical excitation. Adapted from ref 62. (b) Schematic of a phase-change-tunable Au nanodisk–Sb_2_S_3_ cavity metasurface (left). Corresponding reaction yields for methylene blue degradation in the amorphous and crystalline states (right) demonstrate thermally reconfigurable control of photochemical activity. Adapted from ref 113. (c) Schematic of a GaP nanodisk metasurface on a SiO_2_ substrate supporting anapole excitations, enabling strong light confinement within the resonators. Adapted from ref 114. (d) Absorbed power distributions in GaP nanodisks for lattice resonance (LR) and anapole excitation (AE) modes (left). Corresponding absorbance spectra of the metasurface and a Pt-coated film (middle), together with incident photon-to-current conversion efficiency of the photoelectrodes (right), demonstrating enhanced photoconversion enabled by resonant metasurface design. Adapted from ref 115. (e) Measured mid-infrared absorption/emission spectra of the fabricated structure at different temperatures during heating (H) and cooling (C) cycles, highlighting temperature-dependent emissivity and thermal hysteresis. Adapted from ref 116. (f) Absorption spectrum of a CuSO_4_·5H_2_O film (black) and reduction in the dehydration onset temperature (−Δ*T*) for metasurfaces with different resonant modes (blue), demonstrating resonance-assisted control of phase-transition energetics. Adapted from ref 117.

Beyond static thermal optimization, Lyu et al.^113^ demonstrated thermally reconfigurable control of photochemical activity using a phase-change-tunable Au nanodisk–Sb_2_S_3_ cavity metasurface 113. Thermally induced switching between the amorphous and crystalline states modifies the hybridized plasmon–cavity resonance, resulting in reversible modulation of methylene blue degradation yields under identical illumination conditions and demonstrating dynamic control of light-driven chemical reactions within a single metasurface architecture (Figure 5b).

While early work focused largely on plasmonic platforms, dielectric and hybrid metasurfaces are emerging as complementary systems for solar energy conversion and green fuel production. Anapole modes in high-index nanoresonators have been shown to localize electromagnetic energy within subwavelength volumes for controlled nanoscale heat generation^114,119^ (Figure 5c), and Hüttenhofer et al. showed that GaP-based dielectric metasurface photoelectrodes enhance solar water splitting through engineered resonances^115^ (Figure 5d). Despite these advances, systematic correlations between mode-specific energy localization, nanoscale engineering of temperature distributions, and catalytic turnover rates remain an important open challenge.

### Thermal Photonics

Kirchhoff’s law ties emissivity to absorptivity, so the same resonance engineering that concentrates absorption for photothermal heating also governs thermal emission. Thermo-optical tuning therefore provides a natural route to reconfiguring the spectral, directional, and polarization properties of thermally radiated light. Transforming the incoherent, spectrally broad, undirected, and unpolarized thermal radiation of blackbodies into coherent, narrowband, directional, and polarized thermal light^120–126^ is possible by engineering metasurfaces to impart desired properties to emitted light. The TO effect has been incorporated to tune the emission bandwidth,^127^ directionality,^124,128^ amplitude,^129^ and combinations thereof^130^ and has enabled spatial encoding of thermal emission.^131,132^ Temperature-adaptive radiative cooling metasurfaces have modulated emissivity in the atmospheric transparency window for dynamic thermal regulation^116,133,134^ (Figure 5e). Further demonstrated capabilities include tunable chiral IR emission,^135^ polarization conversion,^136^ Huygens’ metasurfaces,^137^ tunable IR filters,^138^ temperature and refractive-index sensors,^139,140^ and TO switches.^141,142^ Further TO reconfigurability is expected to advance applications such as nonreciprocal emission at thermodynamic limits,^143,144^ thermophotovoltaics, near-field radiative heat transfer, thermal camouflage, and building and device heat management.

### Biomolecular Detection

Infrared metasurfaces whose resonances spectrally overlap with molecular vibrational fingerprints can amplify absorption signatures by orders of magnitude; thermo-optical tuning allows these resonances to be swept across an analyte’s absorption spectrum without fabricating multiple devices, enabling broadband molecular identification on a single chip. Reconfigurable noncontact molecular detection has been enabled by enhancing local infrared electromagnetic fields in molecular environments with infrared metasurfaces whose resonances are tuned thermally or electro-thermally.^145,146^ A metasurface-enhanced infrared photothermal technique has further leveraged the TO effect to offer far-field, spatially resolved infrared spectroscopic imaging of localized single-resonator-mediated heating with outstanding biomolecular detection sensitivity.^147^ Moreover, thermal circular dichroism—the temperature difference of a sample when subject to right or left circularly polarized light—has been predicted to significantly enhance chiral molecular detection sensitivity when nanophotonic structures are designed to optimize collective thermal effects.^60^ Thermo-optical tuning of infrared metasurfaces is expected to advance devices for environmental monitoring, medical diagnostics, and chemical analysis.

### Strong Coupling

When a metasurface resonance is spectrally aligned with a molecular vibration or electronic transition and the coupling rate exceeds the loss rates of both constituents, the system enters the strong-coupling regime and forms hybrid light–matter states (polaritons) whose properties differ from those of either constituent. Because thermo-optical tuning can shift a metasurface resonance across a molecular absorption band, it offers a reversible means to switch polariton formation on and off, providing a unique experimental handle to isolate strong-coupling effects from purely thermal contributions to modified reactivity. Metasurfaces can resonantly couple to molecular excitations, and pushing the coupling strength to the extreme yields the electronic or vibrational strong coupling regime. In polaritonic chemistry, optical resonators are strongly coupled to molecular excitations to target perturbations of molecular reaction dynamics. The strong-coupling regime has been associated with modified chemical reaction rates,^148–152^ altered chemical product ratios,^153^ shifted phase transition temperatures,^117,154^ modified morphology,^155^ enhanced ionic conductivity,^156^ and various quantum effects.^157–161^ When the resonator mode is tuned to the molecular excitation, resonant radiative energy transport between the resonator and molecules is thought to provide an additional thermal transport channel that may underlie observed reactivity perturbations^117^ (Figure 5f).

The possibility of switching these chemical reaction perturbations on and off by thermo-optically tuning metasurface modes across molecular absorption bands represents a compelling frontier.^162^ Recent demonstrations of programmable polaritonic photonics showed modulation of the coupling strength by external heating or ultrafast photothermal actuation,^145,163^ and controllable switching of strong-coupling-enabled effects is expected to enable a deeper study of the underlying principles and their potential applications.

## Future Perspective and Challenges

This review has framed thermo-optical metasurfaces as coupled multiphysical systems whose performance is captured by the figures of merit η, $F_{T}$ , and $F_{P}$ (see the “Resonance-Level Description and Figures of Merit” section). The benchmarking in Table S2 reveals how unevenly the design space has been explored and points to several promising directions.

A central challenge is extending control from uniform or single-pixel heating to independently addressable meta-atom arrays. Integrated microheater networks,^71,72^ structured pump beams,^57^ and inverse-designed absorber geometries^62,88^ could enable programmable wavefronts and spatial light modulators whose refresh rates are set by thermal diffusion rather than mechanical inertia. The diffusion length *L*_D_ (eq 3) imposes a fundamental pixel-pitch floor;^2^ lowering it demands reduced substrate diffusivity, faster modulation, or thermal isolation strategies such as suspended membranes.^145^

Speed is the most frequently cited limitation. Thermal time constants of microseconds to milliseconds^57,78^ lag electro-optic alternatives by orders of magnitude, yet thermo-optical actuation requires no transparent electrodes, bias voltage, or doped contacts, preserving the resonance quality and simplifying fabrication. Substrate thinning, heat-sinking nanostructures, and pulsed photothermal drive with transient overshoot^57^ are largely unexplored acceleration routes. Equally important is identifying application regimes, such as adaptive optics, environmental sensing, and display refresh, where microsecond speeds suffice, positioning TO metasurfaces alongside rather than in direct competition with faster but more complex technologies.

On the measurement side, reliable nanothermometry within resonant meta-atoms remains an open problem. Raman thermometry can be systematically perturbed by the very resonance it characterizes, through Purcell-enhanced emission and modified excitation cross sections.^73,77^ Developing artifact-aware calibration protocols or alternative probes such as anti-Stokes luminescence or thermal near-field scanning is essential for validating the coupled models of the “Modeling and Design Trade-Offs” section.

In the nonlinear regime, thermo-optical bistability has been demonstrated from *Q* < 10 to *Q* > 10^3^,^4,66,92^ yet systematic design rules linking resonance parameters to hysteresis width and switching power are still emerging. Combining bistable volatile memory^5^ with PCM nonvolatility^45^ could open routes to neuromorphic photonic computing. Similarly, TO tuning of quantum light sources via SFWM and SPDC is in its infancy;^111^ demonstrating spectral routing of photon pairs through temperature control would bridge metasurface photonics and quantum information processing. For chemistry and spectroscopy, the convergence of photothermal catalysis,^62,118^ surface-enhanced IR sensing,^132,147^ and polaritonic chemistry^117,153^ around a single metasurface platform suggests a closed-loop paradigm: generate designed local temperature fields to control the reaction environment, monitor it spectroscopically through the same resonances, and thermo-optically switch strong-coupling effects on and off^145,162^ to disentangle thermal from nonthermal contributions to modified reactivity.

Finally, translating these demonstrations into practical devices will require thermal packaging, material stability (particularly PCM cycling endurance),^45,50^ CMOS-compatible fabrication, and standardized reporting of η, $F_{T}$ , $F_{P}^{abs}$ , and $F_{P}^{in}$ alongside switching speed and endurance. Thermo-optical metasurfaces therefore occupy a distinctive niche: they combine the design freedom of passive flat optics with thermally programmable actuation that can be implemented through contactless optical heating, integrated electrical heaters, or ambient-temperature variations, depending on the target application. Their most compelling future will not be as direct replacements for the fastest electro-optic or mechanical platforms, but as thermally programmable photonic systems in which heat, optical resonance, and material response are engineered together.

Looking forward, the central opportunity is to turn the apparent liability of absorption into a design principle. If temperature fields can be generated, measured, and patterned with the same precision now routinely applied to optical phase, thermo-optical metasurfaces could evolve from tunable components into multifunctional platforms for adaptive optics, nonlinear and quantum photonics, spectroscopy, and chemistry. In this sense, heat is not merely an unavoidable byproduct of nanophotonic confinement; it is an additional degree of freedom for controlling light–matter interaction at the nanoscale.

## Supplementary Material



## Data Availability

No new experimental data were generated in this Review. The data used to construct the comparative tables and analysis plots are collected from the cited literature and are provided in the Supporting Information. Additional information is available from the corresponding author upon reasonable request.

## Notes

Extended tabulated material parameters and thermo-optical performance metrics, including Table S1, which summarizes representative thermo-optical and thermal properties of material platforms discussed in the paper, and Table S2, which compares representative resonance platforms and photothermal bistability/nonlinear self-action metrics (PDF)
