# Supplementary material for: Thermally Reconfigurable Metasurfaces: From Linear Wavefront Control to Nonlinear and Chemical Functionality
Source: Nano Lett. 2026 Jul 10;26(28):8941–59. doi: 10.1021/acs.nanolett.6c02092 (PMC13397896; doi:10.1021/acs.nanolett.6c02092)
Supplement: Supplementary file 1 [file nl6c02092_si_001.pdf]

Supporting Information

Thermally Reconfigurable Metasurfaces: From Linear Wavefront Control to Nonlinear and Chemical Functionality

Omer Can Karaman[1], Gopal Narmada Naidu[1], Agostino Di Francescantonio[1], Diana Dall'Aglio[1], Elif Nur Dayi[1], Gloria Davidova[1], and Giulia Tagliabue[1,*]

[1]Laboratory of Nanoscience for Energy Technologies (LNET), STI, École Polytechnique Fédérale de Lausanne, 1015 Lausanne, Switzerland

*Corresponding author: giulia.tagliabue@epfl.ch

S1. THERMO-OPTICAL PROPERTIES OF MATERIALS

Table S1. Material-level thermo-optical and thermal parameters for the main material platforms discussed in Section 3. Values are given at one representative wavelength. dn/dT is reported in units of 10^{-6} K^{-1} and dk/dT in K^{-1}. Unless otherwise stated, ρC_p and κ are room-temperature bulk values; thin-film values can differ because of deposition conditions, stoichiometry, porosity, and crystallinity.

Material	Wavelength	n	k	dn/dT (10^{-6} K^{-1})	dk/dT (K^{-1})	ρC_p (MJ m^{-3} K^{-1})	κ (W m^{-1} K^{-1})	Ref.
High-index semiconductors and dielectrics								
a-Si	800 nm	~4.2	~0.06	≈ 400	≈ 6×10^{-5}	~1.6	~1.5	57
c-Si	780 nm	3.695	≈ 0	272	–	1.66	140	12,13
Ge	5 μm	~4.0	≈ 0	416	–	1.72	59.9	12,13
GaAs	1.15 μm	3.475	≈ 0	250	–	1.84	54–55	12,13
AlGaAs	770 nm	3.55	≈ 0	~ 230	–	–	~ 15	20
InP	5 μm	3.080	≈ 0	83	–	~1.5	68–73.5	12,13
SiOC	1550 nm	≈ 2.2	< 10^{-4}	250	–	–	–	17
Si_3N_4	1550 nm	~2.0	≈ 0	25.1 ± 0.8	–	–	33	13,23
SiO_2 / fused silica	1550 nm	~1.44	≈ 0	5.67 ± 0.53	–	1.65	1.4	13,23
TiO_2 (ALD anatase)	1550 nm	~2.3	≈ 0	−230	–	–	–	26
$As_{40}S_{60}$ glass	5 μm	2.407	≈ 0	~ 90	–	–	–	13
PbTe	5 μm	5.857	–	−1500	–	~1.23	2.3	12–14
Polymers, liquid crystals, phase-change materials, and metals								
PMMA	1550 nm	~1.49	≈ 0	−130	–	1.75	0.20	13,53,55
SU-8	1310 nm	~1.58	≈ 0	−187	–	–	~0.2	24
E7 liquid crystal	1550 nm	anisotropic	≈ 0	transition dependent	–	–	–	54
GST ($Ge_2Sb_2Te_5$)	1550 nm	phase dependent	phase dependent	–	–	–	–	50
VO_2	1550 nm	phase dependent	phase dependent	–	–	–	–	52,94
Au	800 nm	~0.15	~4.9	–	thermal change in $\epsilon(\omega, T)$	2.49	317	13,27
Ag	800 nm	~0.04	~5.6	–	thermal change in $\epsilon(\omega, T)$	2.47	429	13,28

S2. EXTENDED SUMMARY OF METASURFACE-LEVEL THERMO-OPTICAL FIGURE OF MERITS

**Resonance**	**Material**	λ_0 **(nm)**	$\Delta\lambda_0$ **(nm)**	Q	η	$\mathcal{F}_T$ **(K^{-1})**	$\mathcal{F}_P^{\text{abs}}$ **(W^{-1})**	$\mathcal{F}_P^{\text{in}}$ **(W^{-1})**
q-BIC	c-Si[67]	1548.99	0.230	7000	1.04	0.348	–	–
	c-Si[68]	1550	20.4	3000	39.5	0.395	–	–
	c-Si[73]	639	2.0	100	0.313	1.20e-3	8.24	7.83
	a-Si[93]	1530	7.7	200	1.01	2.53e-2	$\gtrsim 3.0e1$	1.48
	LN[164]	1168	3.0	300	0.771	9.07e-3	–	–
	Ge/CaF_2[16]	3190	40.0	68	0.852	8.52e-3	–	–
	Ge[87]	1540	25.0	80	1.30	1.05e-2	–	–
Fano (collective ED–MD)	a-Si[89]	784	12.0	78.4	1.20	1.16e-2	–	–
Fabry–Perot (overcoupled)	a-Si[78]	1525	2.70[a]	190	0.336[a]	–	–	9.59e1[a]
Mie	a-Si[57]	800	40.0	30	1.50	3.13e-3	68.9	37.9
	PbTe[14]	7800	1.73	4500	1.00	1.00	3.62e5	3.62e5
Guided mode (QGM)	c-Si[65]	1553.1	0.802	2206	1.14	0.116	9.05	1.81
	c-Si[86]	1480.5	6.0	350	1.42	1.89e-2	–	–
High-Q transmissive (quasi-local/Fano)	a-Si[71]	1534.8	–	3780	–	–	–	–
PCM cavity-loaded	GST225[76]	1538.3	11.7	926	7.04	–	–	–
Plasmonic	Al/PDMS[75]	776	27.4	70	2.47	2.06e-2	–	–
Bistability / nonlinear self-action								
q-BIC (membrane)	c-Si[92]	961[b]	~17[b]	~105[b]	~1.8[b]	~9.7e-3[b]	–	~18[b]
q-BIC (super-cavity)	c-Si (doped)[4]	1400	~66[c]	615	~29[c]	~3.5e-2[c]	–	–
Mie (nanocuboid)	a-Si[66]	740	–[d]	4.4	–[d]	–[d]	–	–
...	...	...	...	...	...	...	...	...

Notes. [a] For[78], $\Delta\lambda_0$ was inferred from the reported tuning slope and π-phase electrical power as $\Delta\lambda_0 \approx (0.77\ \text{nm/mW}) \times (3.5\ \text{mW}) = 2.70$ nm; η and $\mathcal{F}_P^{\text{in}}$ were then computed from this inferred shift. For[71], the paper reports the active operating wavelength and Q for the transmissive high-Q design, but not an explicit thermally induced $\Delta\lambda_0$, so the remaining metric columns are left blank. For[76], $\Delta\lambda_0$ corresponds to the amorphous-to-crystalline resonance displacement; $\mathcal{F}_T$ and power-normalized metrics are not directly comparable to heater-driven thermo-refractive cases and are therefore left blank.

Bistability entries. [b] For[92], values correspond to the bistable regime at $k_{\|}/k_0 = 0.32$: Q is the loaded quality factor extracted from total losses ($\gamma_r = 5.97 \times 10^{12}$ rad/s, $\gamma_{nr} = 3.37 \times 10^{12}$ rad/s); $\Delta\lambda_0 \approx 17$ nm and $\Delta T \approx 186$ K are the maximum resonance shift and temperature rise observed within the hysteresis cycle; $\mathcal{F}_P^{\text{in}}$ uses $P_{\text{in}} \approx 100$ mW (maximum available laser power). These metrics characterize the nonlinear self-action regime and are not directly comparable to the linear tuning entries above. [c] For[4], the design is a doped c-Si ($n_d = 4.6 \times 10^{18}\ \text{cm}^{-3}$) nanocylinder supporting a quasi-BIC super-cavity mode excited by an azimuthal vector beam. Values are from numerical simulation in the linear heating regime ($\Delta T = 815$ K at $I = 0.55\ \text{mW}/\mu\text{m}^2$); $\Delta\lambda_0$ and η are estimated from $\Delta n = n_1 \Delta T$ with $n_1 = 2 \times 10^{-4}\ \text{K}^{-1}$. In the nonlinear regime, heating efficiency at optimal detuning reaches ~1400 K/(mW/μm^2). [d] For[66], the a-Si nanocuboid (155 nm width, 142 nm height) supports a broad Mie resonance with $Q \approx 4.4$. Bistability arises from photothermal competition between absorption and heat dissipation at excitation intensities of ~2–3.6 mW/μm^2, with effective nonlinearity reaching ~100th power of excitation intensity at the transition. The extremely low Q renders $\Delta\lambda_0$-based metrics ill-defined; the relevant figure of merit is the switching intensity contrast rather than spectral shift.

Table S2. Comparison of resonance platforms and representative thermo-optical metrics. $\eta = Q\,|\Delta\lambda_0|/\lambda_0$ is the linewidth-normalized detuning; $\mathcal{F}_T = \eta/\Delta T$; $\mathcal{F}_P^{\text{abs}} = \eta/P_{\text{abs}}$; $\mathcal{F}_P^{\text{in}} = \eta/P_{\text{in}}$.
